# Exploring the acceptability of a brief, rapid-access, self-harm focussed talking therapy: a qualitative analysis of patient experiences

**DOI:** 10.1136/bmjopen-2026-118241

**Published:** 2026-06-03

**Authors:** Caroline Clements, Helen Mulholland, Anna Hunt, Catherine Mills, Kari Kvamme- Mitchell, Naheed Tahir, Cecil Kullu, Peter Taylor, Mark Gabbay, Pooja Saini

**Affiliations:** 1 Division of Psychology & Mental Health, School of Health Sciences, Manchester Academic Health Sciences Centre, The University of Manchester, Manchester, UK; 2 Institute of Population Health Sciences, University of Liverpool, Liverpool, UK; 3 School of Psychology, Liverpool John Moores University, Liverpool, UK; 4 Mersey Care NHS Foundation Trust, Liverpool, UK; 5 Mental Health Liaison Team, Greater Manchester Mental Health NHS Foundation Trust, Manchester, UK

**Keywords:** Primary Care, Patients, Suicide & self-harm, Feasibility Studies

## Abstract

**Objective:**

To explore the acceptability of the Community Outpatient Psychotherapy Engagement Service for Self-harm (COPESS) intervention and trial procedures for participants.

**Design:**

A mixed-method approach and a single-blind randomised controlled trial design with 1:1 allocation to either COPESS plus treatment as usual or treatment as usual alone.

**Setting:**

Primary care.

**Participants:**

Fifteen semistructured interviews were conducted with participants in the COPESS trial; eight were randomised to the intervention arm, and seven were randomised to the treatment-as-usual arm. Interviews lasted 30–60 min.

**Intervention:**

COPESS is a brief, relational psychotherapy delivered over 4 weekly sessions plus a follow-up, focusing on understanding difficult emotional states and their links to self-harm through here-and-now relational and emotional processes.

**Analysis:**

Thematic analysis allowed exploration of themes important to participants in their experiences in the COPESS trial and their experiences of care for self-harm more generally.

**Findings:**

Five themes were identified as associated with the acceptability of the COPESS intervention and trial: self-harm as a primary problem, what I needed when I needed it, being heard and understood, online delivery of the intervention and lasting impacts. Participants generally expressed positive views about the intervention, citing a need for services that specifically target self-harm and an appreciation of the rapid access to help. Strong relationships with the therapist were highly valued and not diminished by online delivery of the intervention. Positive impacts continued post therapy sessions.

**Conclusions:**

These results support the acceptability of the COPESS intervention, the need for self-harm specific services and support moving forward to a full trial.

**Trial registration:**

Pre-registered on clinicaltrials.gov (NCT04191122) on 9 December 2019.

**STRENGTHS AND LIMITATIONS OF THIS STUDY**

- The interview topic guide and thematic analysis were developed with input from patient and public contributors, supporting relevance and acceptability of the qualitative study design.
- Participants from both the intervention and treatment-as-usual arms of the trial were included, allowing for consideration of similarities and differences in experiences across trial conditions.
- Interviews provided in-depth accounts of acceptability, benefits and barriers to engagement with Community Outpatient Psychotherapy Engagement Service for Self-harm, as well as reflecting on other services participants had experienced in the past.
- As a qualitative study embedded within a feasibility trial, the sample was small and drawn from a single geographical area, which may limit the relevance of the themes presented here to other settings.
- The participants in the interview study lacked demographic diversity, with all participants being white and most being younger women, which may limit transferability of themes to other populations.

## Introduction

 Reduction and prevention of self-harm is a major national and international public health priority.^1 2^ Commonly defined as any intentional act of self-injury or self-poisoning regardless of motivation or suicidal intent, self-harm often indicates underlying psychological problems and is strongly associated with suicide and other poor outcomes.^3^,^5^ Over 200 000 self-harm presentations are made to hospital Emergency Departments each year in England with rates in the community estimated to be much higher as survey work indicates the majority of people who self-harm do not attend the emergency department.^6^,^8^ General practitioners (GPs) are the main community primary care providers in England and often a first point of contact for people experiencing mental health problems, including problems related to self-harm. Yet GPs report feeling underprepared when treating people who self-harm.^9^,^12^ Given the distress and social and economic costs incurred by people who self-harm, their friends, family and wider society, providing effective clinical care to reduce the risk of further self-harm is essential.^7^

National Institute for Health and Care Excellence (NICE) guidance for the care of people who self-harm recommends rapid access to talking therapies.^13^ Talking therapies that target underlying psychological processes, such as cognitive behavioural therapy and psychodynamic interpersonal therapy (PIT), have been shown to reduce repetition of self-harm as well as depressive symptoms.^14^,^16^ However, there are questions over whether cessation or reduction of self-harm behaviour is the most appropriate primary outcome measures in efficacy evaluations.^17 18^ Qualitative work with people who have lived experience of self-harm indicates that for some people self-harm serves as a way of avoiding potentially more serious or damaging behaviours and they would potentially not engage with services that prioritise cessation of self-harm over more personally meaningful measures of improvement.^17^ More recent approaches encourage a broader patient-centred view of successful outcomes, such as improvements in general well-being, improved interpersonal relationships, more adaptive responses to stressors and the general acceptability of the intervention to those receiving it and those providing it.^19 20^

The Community Outpatient Psychotherapy Engagement Service for Self-harm (COPESS) is a brief, rapid access talking therapy which tackles individual factors that precipitate self-harm behaviour and related symptoms of depression.^21^ Designed to be implemented in primary care settings, COPESS is a modified version of PIT in combination with cognitive ‘maps’ and goodbye letters typically used in cognitive analytic therapy, previously used in an NHS hospital setting in England.^22^ COPESS was piloted in community settings in a successful feasibility study that looked at recruitment, retention and trial procedures.^21^ Essential to the feasibility evaluation was direct feedback from the participants via interviews, regarding the acceptability of the COPESS therapy as well as the overall trial procedures. Participants allocated to the treatment arm of the trial and participants allocated to treatment-as-usual (TAU) were included in these interviews. The specific aims were to look at (1) the overall acceptability of COPESS, (2) key aspects of COPESS that were important to participants and (3) participants’ previous experiences of seeking care for self-harm and what services for self-harm they would like to receive.

## Methods

### Design

A convergent mixed methods design was used, in which qualitative interviews and quantitative feasibility outcomes were collected during the same study period (see Saini *et al*).^23^ The qualitative component adopted a pragmatic qualitative descriptive approach to understand participant experiences of trial participation across both arms of the trial and inform future trial design. Participants in the intervention arm additionally discussed the acceptability of the COPESS intervention and engagement with the therapy. The COPESS feasibility study tested a randomised controlled trial format in preparation for a full trial and took place in the Northwest of England from February 2020 to September 2022. Fifty-five participants were recruited from GP practices with 28 randomised to the COPESS intervention arm and 27 randomised to receive TAU. The first participant was recruited into the study on the 13th^th^ of November 2020. During the trial consent procedure, participants were asked if they would be willing to take part in one-to-one interviews about their participation and provided formal consent prior to their interview.

### Participants and recruitment

Participant recruitment was via a written information pack through referral from a GP (following consultation for self-harm or via searches of practice records to identify presentations for self-harm issues within the previous 6 months), or self-referral via advertising material within community settings (eg, student counselling services and walk-in centres). Details of recruitment criteria and procedures can be found in Saini *et al*.^21^ Following an expression of interest, a brief screening interview with one of the research team determined eligibility, and preliminary consent was taken. Participants were invited for a further meeting where consent to take part in the trial was confirmed, including consent to take part in interviews. Once trial procedures were completed, participants were invited to a one-to-one interview. Participant consent was reconfirmed before the interview took place. A purposive sample of 15 participants (gender and feasibility trial arm were the main sampling parameters) captured variation in views and experiences of participants. Participant details are shown in table 1. Eight participants from the intervention arm took part. Seven self-identified as female. Five were enrolled students, and most were younger in age (ie, under 30, n=6). Seven participants allocated to the TAU arm of the trial were interviewed. Three self-identified as female. Three were enrolled students, and most were younger in age (ie, aged 16–30, n=5).

**Table 1:** Demographic characteristics and trial arm allocation for interview participants within the COPESS feasibility trial

Trial **arm**	Gender	Age	Ethnicity	Student
COPESS	Female	16–20	White British	Yes
COPESS	Male	51–60	White British	No
COPESS	Female	16–20	White British	Yes
COPESS	Female	16–20	White British	Yes
COPESS	Female	31–40	White British	No
COPESS	Female	21–30	White British	Yes
COPESS	Female	21–30	White British	No
COPESS	Female	16–20	White British	Yes
TAU	Female	16–20	White British	No
TAU	Male	51–60	White British	No
TAU	Male	16–20	White British	Yes
TAU	Female	21–30	White British	No
TAU	Male	51–60	White British	No
TAU	Female	16–20	White British	Yes
TAU	Male	16–20	White British	Yes

COPESS, Community Outpatient Psychotherapy Engagement Service for Self-harm; TAU, treatment-as-usual.

### Data collection

Semistructured interviews were conducted via Microsoft Teams between January 2021 and September 2022 by the study research assistant (RA), who is experienced in qualitative interviewing (AH). The same RA conducted eligibility checks and guided participants through their involvement in the COPESS trial; hence, there was some professional interaction between interviewer and participant before the interview. The COPESS participant information sheet included details of the aims of the COPESS trial and reasons for conducting the study, and the participant may have been primed to the topics of interest to the interviewer. Only the participant and RA were present for the interview. Interviews were audio recorded, transcribed in full by a professional transcription service and anonymised by the RA. Audio-recordings negated the need for field notes either during or following each interview. Interviews lasted between 30 and 60 min. Broadly, the interviews asked open questions, supplemented by prompts, on participants’ views about taking part in the trial, including their motivation for taking part, previous experience with mental health pathways in the NHS, and if they were allocated to the COPESS intervention; the intervention received, benefits of the intervention and challenges to engagement. The topic guide was developed with input from the research team and the project-specific public advisory group.

### Patient and public involvement

The trial included six public and patient members from design and through the duration of the study. Public advisors were members of the public and/or service users with knowledge of COPESS and the locality in which it was delivered. The public advisors were involved in developing data collection materials, recruitment strategies and the analysis strategy. Three public advisors are authors on this paper (CM, KK-M, NT) and have contributed to the analysis of the data, drafting the paper and interpretation of results.

### Thematic analysis

Braun and Clarke’s^24^ reflexive thematic analysis was used as it provides a flexible and systematic approach to identifying patterns across participants’ accounts and can be applied within different epistemological positions.^24^ It was suitable for this study, where a pragmatic approach informed by critical realist principles was taken, to understand participants’ experiences of COPESS while informing intervention and trial refinement in a feasibility trial context. Analyses were conducted in parallel with ongoing recruitment with sampling informed by feasibility trial parameters and the richness of accounts. Paper-based interview transcripts were analysed manually.^25^,^27^ Transcripts were read repeatedly to support familiarisation with the data. Initial codes were generated inductively to capture meaningful features of participants’ accounts. Codes were iteratively reviewed, refined and compared within and across transcripts through ongoing reflexive discussion and engagement with the dataset. Codes were then developed into candidate themes, which were reviewed and refined to ensure coherence, distinctiveness and fit with the data before being defined and named. This iterative and reflexive approach supported a transparent and systematic analysis of participants’ experiences. To limit the possible bias of a single-analyst perspective, multiple members of the research team (AH, PS, CC, HM, CM) examined a sample of transcripts to compare their initial perceptions of the interview data and discuss initial codes, which were compiled into a word document and arranged into a preliminary thematic structure. This was repeated with additional transcripts as they became available, in an iterative process, with codes and themes being discussed and refined further until consensus was reached. The thematic structure was presented to the multidisciplinary research team for further comment and feedback, before being finalised by a subgroup of the research team (CC, HM, PT, PS).

## Results

There were commonalities of experience and opinion within participants’ accounts that tapped into important issues around care for self-harm beyond those directly related to the COPESS trial. Across repeated analysis and review, participants’ thoughts and experiences coalesced around five main themes: *self-harm as a primary problem, what I needed when I needed it, being heard and understood, online delivery of the intervention* and *lasting impacts*.

### Self-harm as a primary problem

The COPESS intervention focus on self-harm was valued by participants, who sought conversations to help deal with significant feelings underpinning their self-harm, rather than medication to mask those feelings. This was contrasted with previous experiences of seeking help for self-harm, particularly from primary care services, often being referred to general treatment for mental health problems, such as depression. Participants indicated these were secondary intervention targets for them in comparison to self-harm.

Yes, they (GPs) just try and shoo you away, I feel, with some tablets and that is it. No one has a conversation with you or anything, about how you are feeling. [1103, TAU]

It is more focused on just general depression, anxiety. Self-harm is regarded as one question on your recent questionnaire to tick off, as opposed to being something that can be quite significant and quite a main thread through someone’s mental health struggle. (1027, TAU)

Where referral to psychological services was available, the expectation of long waiting times discouraged participants from engaging. The lack of immediate support left them feeling undervalued by clinical services.

I have had conversations with previous GPs over the years, but I have never really gone through anything, because the wait lists are so long. It is sometimes like, “Oh, what is the point? I am going to be waiting 3 years. (1012, COPESS)

Many participants reported being unaware of any specific services for self-harm within primary healthcare and expressed disappointment that self-harm was not acknowledged as a significant problem in itself.

People are just self-harming but there’s no stuff out there to do with self-harm. And it’s [self-harm] just getting ignored a lot, we need to look at that more. […] They’ve (clinical services) tried to do counselling and that, but I’ve never sat down and had therapy for self-harming. Which is wrong, I’ve not heard of therapy for self-harming to be honest, no, nothing that I’ve heard of. (3008, TAU)

This common struggle to get help for self-harm was contrasted with an appreciation of the COPESS intervention being offered.

I’ve been going through this for about 6 years now, self-harming, and then to have like something so specified come along to help you get to the root of the problem, it was crazy. (1001, COPESS)

Participants view self-harm as ‘significant’ in its own right and seek ‘a conversation’ about what underpins their self-harm. The participants’ positive view of COPESS and the need for specialist self-harm services was evident regardless of the broader mental health picture for an individual.

### What I needed when I needed it

Many participants reflected positively on how quickly they were able to access the intervention. As mentioned previously, long waiting lists for psychological interventions discouraged engagement with services and reflect an inability in current care pathways to respond to the time-sensitive nature of self-harm.

So, when I needed the help, I couldn’t get the help that I actually needed, and by the time I actually got the help that I needed about 6 months prior to that, it didn’t benefit me as much as it did this time because I got it near enough straightaway, which was what I needed and when I needed it… (5002, COPESS)

I think, for me actually, it came at the right time and it has really helped me make progress. Because the thought of still being back on a waiting list now… It puts a lot of people off. (1102, COPESS)

Some participants were sceptical about what could be achieved in five therapeutic sessions. But the focused nature of the sessions and the aims developed in collaboration between the patient and therapist enabled positive patient-led conversations and outcomes.

I think it was good actually, the length. I feel like there was enough time to get into it. But I have had therapies where sometimes it feels like it is dragging out towards the end, but this one didn’t feel like that. (3007, COPESS)

It feels like I’ve covered so much and talked, and it’s been going for a long time, but actually, it was only a handful of sessions spread over a long period of time, which is crazy. (1001, COPESS)

Some participants expressed they would have benefited from further sessions, but they were still able to see progress in addressing their self-harm.

Maybe I could have done with a little bit more. But in my head now, I’ve got that confidence that I know that it has worked. And it’s a starting block for me, that I know where to go from this now, should I feel that I need to. (1102, COPESS)

Overall, the theme of ‘what I needed when I needed it’—with the title taken directly from a participants’ account—presents some confirmation that brief, rapid accessed interventions are both wanted and valued by participants. Even where participants were not allocated to the intervention arm of the trial, they were supportive of the idea of short, focused interventions.

If you can use that time really productively, if you can focus on getting a toolbox of skills that you can make use of, and something that you can refer back to again and again, it doesn’t matter if you are having less sessions. (1027, TAU)

### Being heard and understood

All participants allocated to the COPESS arm of the trial described it as a positive experience. The active engagement and interest of the therapist was particularly valued. Therapists checking shared understanding of issues participants had raised seemed particularly helpful in developing strong therapeutic relationships. Participants pointed to the importance of being ‘heard’ or ‘listened to’ as an important factor.

I think because I had built what I felt was quite a strong relationship with the counsellor, I was able to be open see what she was getting out of it. And because what she was coming back with was what I agreed with, that worked for me. (1102, COPESS)

So that has been a big help for me, to be able to talk and explain my problems, having someone who was really good and listening to me and gave me time to explain how I felt. (1017, COPESS)

It was really good. It just felt like she really, really understood me and she always had, like I said, very specific, very impactful questions. […] I felt understood. I felt heard. Yes, it was great. (1001, COPESS)

The therapeutic aims across the COPESS sessions are determined by the participant which may underpin participants’ perceptions of being heard. Facilitating a collaborative focus on patient-led outcomes, those most relevant to them at that time, rather than traditional service-led outcomes, such as self-harm cessation.

[…] it’s also very nice to know that you don’t go into a therapy with someone being like, “This is how you stop and you want to stop right now and that’s how you do it. (1001, COPESS)

This theme describes the importance of being heard and understood for people who self-harm, in contrast to accounts of previous negative experiences of healthcare services, which failed to meet service users’ needs. Being heard and understood reflected a shared, collaborative experience across the therapy sessions, which were central to the positive experience of participants who received the COPESS intervention.

### In-person versus virtual delivery of Community Outpatient Psychotherapy Engagement Service for Self-harm

The COPESS feasibility trial took place during the COVID-19 pandemic. Originally designed to be delivered in-person, sessions were changed to online delivery due to social distancing protocols enforced at that time. This change was discussed in follow-up interviews, and participants’ opinions were mixed about whether online or in-person therapy was preferable. However, participants were generally happy with their experience of the online delivery of COPESS.

I just think for me personally I’d enjoy it online more. But I can completely see why meeting up would be good as well. Everything is online now, isn’t it? (3007, COPESS)

I don’t believe that it being virtual took anything away from it. (1004, COPESS)

Online delivery was seen as beneficial due to being in a familiar and comfortable environment during and particularly after the sessions, suggesting a need for reflection post-therapy, before re-engaging in the flow of participants’ day-to-day lives.

I preferred being at home because I could be just like in my own element, and then just have the therapy, and then reflect on it in my own room, and then I can go back to normal life. (5002, COPESS)

Others cited more practical reasons for preferring online delivery.

But I think it just sometimes works better to fit into my routine if it is from home. (3007, COPESS)

While all participants accepted the necessity of the change to online delivery, some expressed a preference for face-to-face delivery if it had been available. In-person therapy was seen as having the potential to facilitate communication and deepen the therapeutic relationship.

When you’re looking at somebody who’s in a lot of stress or a lot of pain or… You can see it. (1104, TAU)

Probably be different with some people, because some people don’t know how to express how they feel. But with me, yeah face to face would have been good. (3008, TAU)

The preference for online or in-person delivery of the therapy varied based on participants’ individual preferences and circumstances, but the option to have online delivery was broadly supported.

I do definitely think the virtual aspect is a thing worth having, because obviously, some people have mobility issues, and some people will just get so stressed out leaving. I think that aspect alone is a good option to have permanently. (1004, COPESS)

These contrasting views support the importance of patient choice in how the intervention is delivered, while adding support for the legitimacy of virtual therapy sessions.

### Lasting impacts of the Community Outpatient Psychotherapy Engagement Service for Self-harm intervention

Although this was not an efficacy trial of the COPESS intervention, many participants who received COPESS described perceived reductions in their self-harm behaviour and reported that these benefits lasted after completing the sessions.

But outside of the sessions, I carried that information that we covered, like, everywhere with me. And it, sort of, feels like armour at that point, it feels like you’re stronger. It feels like a protective layer around you, like, “I have this knowledge and I know what’s going on now”. (1001, COPESS)

You will see on there (questionnaire measure) that I have not had any even urges or even thoughts about any self-harming behaviours, which that actually gets me emotional thinking about it because it has literally been a constant for the last 11 years. (1012, COPESS)

The use of therapy tools such as visual maps and goodbye letters was perceived as helpful in carrying forward learning from the sessions.

When I’m struggling now, I do find myself referring back to that map. I think, “Right, this is where it links in, and we’ve said this is the exit strategy from this point, so I’ve got it there. (1102, COPESS)

Then I got the diagram that she made up with clouds all around and arrows. All I have to do is look at that if I need any more advice. (4002, COPESS)

Visual maps were sometimes shared with other people in the participants’ support network to share understanding of their self-harm behaviour.

I showed (their girlfriend) that map and she was like, “What?” But now I am like, “I am at this point” and she is like, “Okay, well you know your exits, let’s go (use the exit strategy)*.* (1012, COPESS)

Participants described how they worked with clinicians to identify ‘exits’ so the participant could recognise the patterns linking situations, thoughts, and behaviours and take action to stop these escalating into self-harm. Exits were included in participants’ maps to provide a comprehensive visual aid outside of the therapeutic context. Participants described these tools as helping them develop a sense of control by making sense of pathways and potential exits, leading to self-harm.

It was really useful to draw the map together […] having things put on paper in such a visual way, that was helping, having things written down and feeling that everything was a bit more organised. (P1017, COPESS)

I do think it is helpful because it sums up everything you talked about or went through, so that you’ve got something with you. (3007, COPESS)

Goodbye letters provided a written summary of participants’ therapeutic journey to show the awareness and learning participants have achieved across the therapy sessions, culminating in a written description of the understanding depicted in participants’ individual map. These are written by the therapist and read aloud during the final therapy session. Participants were also invited to write a letter to their therapist or provide this feedback in a medium of their choice to share with the therapist during the final session. However, there is no obligation for the participant to do so. The goodbye letters were generally described positively, and some participants found it a helpful way to draw the therapy to a close. The letters also provided an additional tangible support to participants in their ongoing journey post-therapy.

It was quite a surreal experience to hear it written down, somebody who actively tried, and as far as I could figure, almost entirely understands me. So, that was a nice way to end this, and I know that I’ve got the letter as well if I needed it, which is quite nice. (P1004)

While participants were generally positive about the impact COPESS had on their well-being and/or self-harm behaviour, some acknowledged the emotional impact that conversations during sessions had on them. For many, the COPESS intervention was often described as a helpful starting point in a longer journey of self-harm management and recovery.

I finished the session and I just collapsed for the day, I found it very, very hard going. But what I got out of it has really benefitted me. (1102, COPESS)

I do not want it to end, but it is fine. We can still… It is a step in the right direction, and I can still progress from it. It just will not be the same person or therapy, kind of thing. But it has not disheartened me. (P1012)

## Discussion

This interview study was embedded within an evaluation of the feasibility and acceptability of delivering the COPESS intervention in a potential future full-scale trial. Themes identified, self-harm as a primary problem, what I needed when I needed it, being heard and understood, online delivery of the intervention and lasting impacts, are consistent with broader work on self-harm and suggest that COPESS may address some of these factors. Feedback from participants was consistently positive and provides support for the acceptability of the COPESS intervention and the broader COPESS trial methodology.

The focus on ‘self-harm as a primary problem’ was seen as extremely positive to participants. NICE guidance recommends tailoring interventions to meet the needs of people who self-harm and the accounts presented here support these recommendations. Confirming that participants in the current and previous studies described valuing interventions specifically designed to target self-harm behaviour.^13^ Participants recounted instances of help-seeking for their self-harm behaviour, particularly from primary care, only to be diverted towards care for underlying mental health problems. This fits with evidence that GPs lack knowledge of how to support people who self-harm and have limited referral options.^11 12^ People with lived experience of self-harm highlight a need to understand the underlying reasons for their self-harm and to develop positive coping strategies to engender personal resilience and avert emotional crises.^28^ COPESS was designed to address these needs, and participants’ accounts suggest it does. Further, these needs can only be addressed via improved awareness, insight and action. Participants often described a preference for collaborative psychotherapeutic support over pharmacological focused responses.

In line with the recent recommendations by NICE, COPESS was intended to provide support at the point of need for participants. COPESS was designed as a brief therapy to be accessed quickly. Although some participants initially expressed scepticism of the brief nature of the intervention, many were happy with how much therapeutic work they were able to do within the five sessions to immediately and directly address their self-harm behaviour, getting quickly to the root of the problem. These accounts are consistent with previous work that suggests positive impacts on suicidal behaviours from short or time-limited interventions, such as brief psychological therapies or safety-planning type interventions.^29 30^ Further, both the aims of the COPESS therapy sessions and their outcomes are set by the patient, in contrast to traditional service-led aims and outcomes, which may help facilitate collaborative therapeutic relationships between patients and their therapist. A systematic review of the relationship between the therapeutic alliance and suicidal behaviours concluded that a stronger therapeutic alliance predicted reductions in self-harm behaviour, and this finding is supported in participants’ accounts following engagement in COPESS.^31^ Further, Pascual-Leone and colleagues suggest improved patient outcomes when therapists support patients to engage with deep-rooted emotions.^32^ Collaboratively recognising and sitting with difficult emotions is a key element of the COPESS therapeutic model to engender enhanced emotional processing and subsequent self-management. This may be one possible explanation for the enduring benefits described by some participants.

Recent qualitative research highlights that individuals who seek care following self-harm often emphasise the importance of feeling listened to, validated and treated with compassion, with negative care experiences shaping future help-seeking and engagement with services.^33 34^ Studies exploring brief psychological and crisis-based interventions also suggest that perceived acceptability and engagement are closely linked to relational factors, such as trust, collaborative working and continuity of care.^35 36^ These findings provide important context for interpreting participants’ accounts in the present feasibility study and reinforce the centrality of patient experience in evaluating interventions for self-harm.

Participants reported that the intervention was helpful in reducing their difficulties with self-harm. Such findings need to be treated with caution given they reflect participants’ personal perceptions and explanations of change, and it may be that other factors better explain the improvements participants described. Although not unique to COPESS, participants commented on the benefits they felt they had derived from the maps and goodbye letters used in the COPESS intervention. These were seen as valuable resources during the sessions and beyond to maintain awareness and understanding and support personal resilience building in participants, which is consistent with existing literature.^37 38^ Some participants also used these as a way to help family and friends gain deeper understanding of their self-harm, potentially increasing the ability of the family member to provide support to the participant. Involving family and friends in the care and support of individuals who self-harm is recommended within global and national suicide prevention policies and care guidelines.^2 13^ The advantages of using materials such as maps and goodbye letters to engage family and friends and to promote understanding of and support for self-harm warrant examination via further research.^13^

Online delivery was unavoidable due to the COVID-19 pandemic, but generally participants expressed positive views about online delivery, being more convenient and in a familiar and comfortable environment both during and after the sessions.^39 40^ Indeed, participants alluded to the emotional cost of engaging in COPESS and the need for reflection and recovery before re-engaging with their usual flow of life. Online delivery meant that post-therapy participants could reflect and emotionally recover in the comfort of their own home. Conversely, concerns were also raised over potential issues with technology and the potential for this to disrupt the creation of a strong therapeutic relationship. Offering patient choice about the medium in which their therapy is provided, either in-person or virtually, was advocated by participants in this sample and reflects the recommendation in current NHS policy to facilitate patient choice in the delivery of their care to enhance therapeutic engagement and retention rates.^2^

## Strengths and limitations

This study explored the perspectives and experiences of people with lived experience of self-harm who took part in a feasibility trial of a brief psychological intervention providing personal context to issues around care for self-harm. An interdisciplinary team including people with lived experience of self-harm brought diverse perspectives and increased trustworthiness of the analysis. The topic guide was co-produced with patient and public involvement to ensure suitability and relevance to participants. As this study was embedded within the COPESS trial, the participants who took part may be different from other people who self-harm (eg, people who did not seek help for self-harm). The small sample tended to be younger and included more women than men, and participants were White British, which represents a significant limitation. Race, ethnicity, age and gender may shape experiences of psychotherapy, including perceptions of acceptability, engagement and therapeutic fit.^41 42^ The absence of racial and ethnic diversity may limit the transferability of the findings to more diverse populations, and the results should be interpreted with caution. Future research should prioritise inclusive and targeted recruitment strategies to support participation from racially and ethnically diverse groups and to examine whether experiences of the intervention and trial procedures differ across groups. Gender and feasibility trial arm were the main parameters used for purposive sampling; however, more women than men were recruited into the trial, and more women agreed to take part in the interviews. The study took place during the COVID-19 lockdown period, which necessitated a move to online delivery of the intervention. It therefore remains unknown if patients’ experiences would be the same if delivery had been face-to-face, although studies using the same intervention in other face-to-face settings suggest a similarly positive response among participants.^22^

The interviewer had prior contact with participants through trial research procedures, including eligibility checks and support with trial assessment forms, but was not involved in the delivery of the intervention. Although this contact may have influenced participants’ responses, potential bias was mitigated using a semistructured topic guide, reflexive analytic practices and the clear separation of qualitative interviewing from intervention delivery.^43^ Including participants from both the COPESS and TAU arms provided contextual insight into trial participation across the study. Interviewing participants allocated to treatment as usual also enabled exploration of any disappointment, frustration or other negative experiences associated with not receiving the intervention. Including both groups allowed experiences common to both arms, such as recruitment procedures, to be considered along with experiences specific to the COPESS intervention. This was intended to inform interpretation within a feasibility context rather than to provide a comparison of outcomes. Participant accounts in the intervention arm were predominantly positive, with relatively limited reporting of negative or neutral experiences. Although this may reflect good acceptability, the potential influence of social desirability bias and the interviewer-participant relationship cannot be ruled out.^44 45^ The intervention was delivered by COPESS therapists who were not involved in the qualitative interviews or analysis, and interviews were conducted independently of therapy provision. However, these factors do not eliminate the possibility of response bias, and this should be taken into account when interpreting the findings.

## Clinical implications

Participant interviews and the broad themes they contain suggest that COPESS was acceptable to participants interviewed in this study, as a self-harm specific therapeutic intervention, and may help address unmet needs within current service provision within primary healthcare services. COPESS was also viewed positively by participants in both arms of the trial, with those in the intervention arm frequently citing lasting positive impacts and perceived reductions in self-harm behaviour.

Themes identified in the participants’ interviews have implications for practice in emphasising the need for quick referrals to self-harm specific psychotherapeutic services and the importance of successful management of expectations within brief interventions, as well as indicating the potential for improved engagement where participants can choose the delivery method of the intervention. Further, clinical services would benefit from appreciating and accommodating the need for personal reflection and emotional recovery post-therapy to enhance patients’ engagement and experience of therapy. Study findings suggest that COPESS may be feasible and acceptable for delivery in primary care settings and warrant further evaluation in a full efficacy trial.

## Data Availability

No data are available.
