# Supplementary material for: Exploring the acceptability of a brief, rapid-access, self-harm focussed talking therapy: a qualitative analysis of patient experiences
Source: BMJ Open. 2026 Jun 3;16(6):e118241. doi: 10.1136/bmjopen-2026-118241 (PMC13239522; doi:10.1136/bmjopen-2026-118241)
Supplement: Supplementary file 1 [file bmjopen-2026-118241.reviewer_comments.pdf]

PEER REVIEW HISTORY

BMJ Open publishes all reviews undertaken for accepted manuscripts. Reviewers are asked to complete a checklist review form and are provided with free text boxes to elaborate on their assessment. These free text comments are reproduced below.

ARTICLE DETAILS

Title (Provisional)

Exploring the acceptability of a brief, rapid-access, self-harm focussed talking therapy: a qualitative analysis of patient experiences

Authors

Clements, Caroline; Mulholland, Helen; Hunt, Anna; Mills, Catherine; Kvamme-Mitchell, Kari; Tahir, Naheed; Kullu, Cecil; Taylor, Peter; Gabbay, Mark; Saini, Pooja

---

VERSION 1 - REVIEW

**Reviewer**	**1**
**Name**	**Furqan, Zainab**
**Affiliation**	**University of Toronto, Department of Psychiatry**
**Date**	**18-Mar-2026**
**COI**	**None**

This is an important area of study, and I appreciate the authors' decision to incorporate qualitative methods within a feasibility trial. Overall, the manuscript is well-written and easy to follow. There are, however, some gaps related to methodology that are critical to address.

The authors do not provide a methodological framework that was used to design the qualitative portion of this study- under methods, they only mention that some participants were asked to participate in interviews. This, however, does not give the reader any information about the type of mixed methods approach that was used (sequential vs. convergent and method of integration) or the methodological orientation that was used to guide qualitative data collection and analysis. In the data analysis section, the authors mention thematic analysis by Braun and Clarke- I would like to see some indication of why this approach was chosen.

Lastly, under 'limitations', authors mention that all participants were white- this is a major limitation and needs to be discussed further, given how race and ethnicity can impact participant experiences of psychotherapy.

**Reviewer**	**2**

**Name**	**Hultsjo, Sally**
**Affiliation**	**Ryhov County Hospital, Ryhov County Hospital**
**Date**	**13-Apr-2026**
**COI**	**None**

Thank you for the opportunity to review this article.

This manuscript presents a qualitative interview study embedded within a feasibility randomised controlled trial of the COPESS intervention for individuals who self-harm. The topic is important, timely, and highly relevant to primary care and mental health service provision. The integration of qualitative data within a feasibility trial is a clear strength, and the inclusion of patient and public involvement (PPI) throughout the research process is commendable.

The manuscript is generally well written, and the themes identified are clinically meaningful. This is a valuable and relevant qualitative study that contributes to understanding the acceptability of a brief psychological intervention for self-harm in primary care. However, the manuscript would be strengthened by greater methodological transparency, deeper reflexive engagement, and a more cautious and balanced interpretation of the findings.

Below are some recommendations that I believe could further develop the manuscript.

- The findings from participants in the COPESS arm are overwhelmingly positive, with very limited exploration of negative or neutral experiences. This raises concerns about potential bias, including social desirability bias and the influence of the interviewer-participant relationship. (Clarify whether any contradictory or less positive accounts were identified. Reflect on how the study design (e.g., interviewer involvement in the trial) may have influenced participants' responses).
- The manuscript acknowledges that the interviewer had prior contact with participants; however, this is not sufficiently explored in terms of its implications for data collection and interpretation. (Steps taken to mitigate potential bias)
- while thematic analysis is described, the methodological approach lacks sufficient detail and transparency. (The coding process is described in broad terms but lacks detail on how codes were developed, refined, and applied).
- Although the authors state that this is not an efficacy study, there are several instances where participant-reported reductions in self-harm are discussed in ways that could be interpreted as evidence of effectiveness. (Clearly distinguish between perceived benefit and demonstrated efficacy. It may be helpful to avoid implying causal conclusions based on qualitative data).
- The inclusion of participants from both the intervention (COPESS) and TAU arms is appropriate and potentially valuable in a feasibility and acceptability study, as it allows for contextual comparison with usual care. However, the rationale for including both groups could be more clearly articulated, and it is not fully evident how this comparative design has been utilised in the analysis. The manuscript would benefit from a clearer justification of why both groups were included, as well as a more explicit consideration of whether and how experiences differed between them.

- The sample is relatively small and lacks diversity (predominantly young, female, and White British participants). While this is acknowledged, the implications for transferability should be discussed in greater depth.
- The manuscript would benefit from the inclusion of more recent qualitative research (2022–2025) focusing on patients' experiences of seeking care for self-harm, as well as factors influencing acceptability and engagement with services. While the current references provide a strong epidemiological foundation, they place less emphasis on patient perspectives, which are central to the stated aims of the study. Incorporating up-to-date qualitative and patient-centered studies would strengthen the contextualization of the findings and improve alignment with the study's objectives.

VERSION 1 - AUTHOR RESPONSE

Reviewer: 1

Comments to the Author:

4. This is an important area of study, and I appreciate the authors' decision to incorporate qualitative methods within a feasibility trial. Overall, the manuscript is well-written and easy to follow. There are, however, some gaps related to methodology that are critical to address.

We thank the reviewer for their positive feedback and have addressed the comments regarding the gaps related to methodology throughout the paper.

5. The authors do not provide a methodological framework that was used to design the qualitative portion of this study- under methods, they only mention that some participants were asked to participate in interviews. This, however, does not give the reader any information about the type of mixed methods approach that was used (sequential vs. convergent and method of integration) or the methodological orientation that was used to guide qualitative data collection and analysis.

We have added more information on the mixed methods approach [Page 5].

> *"A convergent mixed methods design was used, in which qualitative interviews and quantitative feasibility outcomes were collected during the same study period (see Saini et al 2025).23 The qualitative component adopted a pragmatic qualitative descriptive approach to understand participant experiences of trial participation, across both arms of the trial, and inform future trial design. Participants in the intervention arm additionally discussed the acceptability of the COPESS intervention and engagement with the therapy. The COPESS feasibility study tested a randomised controlled trial format, in preparation for a full trial, and took place in the Northwest of England from February 2020 to September 2022."*

6. In the data analysis section, the authors mention thematic analysis by Braun and Clarke- I would like to see some indication of why this approach was chosen.

We have now added the following clarification to the Methods section [Page 7]:

“Braun and Clarke’s (2019) reflexive thematic analysis was used as it provides a flexible and systematic approach to identifying patterns across participants’ accounts and can be applied within different epistemological positions. It was suitable for this study, where a pragmatic approach informed by critical realist principles was taken, to understand participants’ experiences of COPESS while informing intervention and trial refinement in a feasibility trial context.”

7. Lastly, under 'limitations', authors mention that all participants were white- this is a major limitation and needs to be discussed further, given how race and ethnicity can impact participant experiences of psychotherapy.

We agree that the lack of racial and ethnic diversity is a significant limitation. We have expanded the limitations section to more fully discuss how race and ethnicity may shape experiences and acceptability of psychotherapy, and we acknowledge that our findings may have limited transferability beyond the study sample. We also note the importance of more inclusive recruitment in future. We have added the following text [Page 16-17]:

“The small sample tended to be younger and included more women than men, and participants were White British, which represents a significant limitation. Race, ethnicity, age, and gender may shape experiences of psychotherapy, including perceptions of acceptability, engagement, and therapeutic fit.37,38 The absence of racial and ethnic diversity may limit the transferability of the findings to more diverse populations and the results should be interpreted with caution. Future research should prioritise inclusive and targeted recruitment strategies to support participation from racially and ethnically diverse groups and to examine whether experiences of the intervention and trial procedures differ across groups.”

Reviewer: 2
Comments to the Author:
Thank you for the opportunity to review this article.

8. This manuscript presents a qualitative interview study embedded within a feasibility randomised controlled trial of the COPESS intervention for individuals who self-harm. The topic is important, timely, and highly relevant to primary care and mental health service provision. The integration of qualitative data within a feasibility trial is a clear strength, and the inclusion of patient and public involvement (PPI) throughout the research process is commendable.

We thank the reviewer for this positive feedback and for recognising importance and relevance of this topic. We also appreciate their acknowledgement of the strengths of embedding qualitative work within the feasibility trial, as well as the meaningful involvement of patient and public contributors throughout the study.

9. The manuscript is generally well written, and the themes identified are clinically meaningful. This is a valuable and relevant qualitative study that contributes to understanding the acceptability of a brief psychological intervention for self-harm in primary care. However, the

manuscript would be strengthened by greater methodological transparency, deeper reflexive engagement, and a more cautious and balanced interpretation of the findings.

We thank the reviewer for this feedback on the methodological transparency, reflexivity, and the interpretation of findings. We have now addressed each of these points in the revised manuscript and have given further details in response to the points below.

Below are some recommendations that I believe could further develop the manuscript.
10. • The findings from participants in the COPESS arm are overwhelmingly positive, with very limited exploration of negative or neutral experiences. This raises concerns about potential bias, including social desirability bias and the influence of the interviewer-participant relationship. (Clarify whether any contradictory or less positive accounts were identified. Reflect on how the study design (e.g., interviewer involvement in the trial) may have influenced participants' responses).

We acknowledge the reviewer's concern regarding the predominance of positive accounts among participants in the COPESS arm and the potential influence of social desirability bias. While some neutral or minor challenges were identified, overall feedback was largely positive, and we have now clarified this in the manuscript. We have also expanded the limitations section to reflect the possibility of influence of the interviewer-participant relationship. Although the intervention was delivered by COPESS therapists who were not involved in data collection or analysis, and the interviews were conducted independently of intervention delivery, we recognise these factors do not remove the possibility of response bias. We have added further reflection on these issues to the limitations section [Page 17]:

"Participant accounts in the intervention arm were predominantly positive, with relatively limited reporting of negative or neutral experiences. Although this may reflect good acceptability, the potential influence of social desirability bias and the interviewer-participant relationship cannot be ruled out.39,40 The intervention was delivered by COPESS therapists who were not involved in the qualitative interviews or analysis, and interviews were conducted independently of therapy provision. However, these factors do not eliminate the possibility of response bias and this should be taken into account when interpreting the findings."

11. • The manuscript acknowledges that the interviewer had prior contact with participants; however, this is not sufficiently explored in terms of its implications for data collection and interpretation. (Steps taken to mitigate potential bias)

We thank the reviewer for this important point. We have expanded the limitations section to clarify the nature of the interviewer's prior contact with participants and its possible implications for data collection and interpretation. We now state that the interviewer had prior contact through research activities and not via intervention delivery. We have also clarified the steps taken to mitigate potential bias, including the use of semi-structured interviews, reflexive analytic practices, and separation of research interview s from intervention delivery [Page 17].

> *"The interviewer had prior contact with participants through trial research procedures, including eligibility checks and support with trail assessment forms, but was not*

involved in the delivery of the intervention. Although this contact may have influenced participants' responses, potential bias was mitigated using a semi structured topic guide, reflexive analytic practices, and the clear separation of qualitative interviewing from intervention delivery."

12. • while thematic analysis is described, the methodological approach lacks sufficient detail and transparency. (The coding process is described in broad terms but lacks detail on how codes were developed, refined, and applied).

We have revised the Methods section to provide greater transparency of the analytic process, including a clearer description of how initial codes were generated, iteratively refined through reflexive discussion, and developed into themes in line with Braun and Clarke's reflexive thematic analysis [Page 7].

"Transcripts were read repeatedly to support familiarisation with the data. Initial codes were generated inductively to capture meaningful features of participants' accounts. Codes were iteratively reviewed, refined, and compared within and across transcripts through ongoing reflexive discussion and engagement with the dataset. Codes were then developed into candidate themes, which were reviewed and refined to ensure coherence, distinctiveness, and fit with the data before being defined and named. This iterative and reflexive approach supported a transparent and systematic analysis of participants' experiences."

13. • Although the authors state that this is not an efficacy study, there are several instances where participant-reported reductions in self-harm are discussed in ways that could be interpreted as evidence of effectiveness. (Clearly distinguish between perceived benefit and demonstrated efficacy. It may be helpful to avoid implying causal conclusions based on qualitative data).

We agree with the reviewer and have revised the manuscript throughout to more clearly distinguish perceived benefits from demonstrated efficacy. References to reductions in self-harm have been reframed as self-reported experiences or perceived changes described by participants, rather than evidence of intervention effectiveness. This has been updated throughout the results and discussion consistent with the feasibility aims of the study.

14. • The inclusion of participants from both the intervention (COPESS) and TAU arms is appropriate and potentially valuable in a feasibility and acceptability study, as it allows for contextual comparison with usual care. However, the rationale for including both groups could be more clearly articulated, and it is not fully evident how this comparative design has been utilised in the analysis. The manuscript would benefit from a clearer justification of why both groups were included, as well as a more explicit consideration of whether and how experiences differed between them.

We have clarified the rationale for including participants from both the COPESS and treatment-as-usual arms. Including both groups enabled experiences of trial participation common to both arms to be explored, while also allowing intervention-specific experiences of COPESS to be identified. We have revised the manuscript to make clearer that this provided contextual understanding of acceptability within a feasibility study, rather than a comparison of outcomes [Page 17].

> *"Including participants from both the COPESS and treatment as usual arms provided contextual insight into trial participation across the study. Interviewing participants allocated to treatment as usual also enabled exploration of any disappointment, frustration, or other negative experiences associated with not receiving the intervention. Including both groups allowed experiences common to both arms, such as recruitment procedures, to be considered along with experiences specific to the COPESS intervention. This was intended to inform interpretation within a feasibility context rather than to provide a comparison of outcomes."*

15. • The sample is relatively small and lacks diversity (predominantly young, female, and White British participants). While this is acknowledged, the implications for transferability should be discussed in greater depth.

We acknowledge the reviewer's concern and have expanded the discussion of transferability. Although the sample size was appropriate for a qualitative study embedded in a feasibility trial, participants were relatively homogeneous. We have now clarified that the findings might be most applicable to populations with similar characteristics and should be interpreted cautiously. The following has been added to the discussion section [Page 16-17]:

> *"The small sample tended to be younger and included more women than men, and participants were White British, which represents a significant limitation. Race, ethnicity, age, and gender may shape experiences of psychotherapy, including perceptions of acceptability, engagement, and therapeutic fit.37,38 The absence of racial and ethnic diversity may limit the transferability of the findings to more diverse populations and the results should be interpreted with caution. Future research should prioritise inclusive and targeted recruitment strategies to support participation from racially and ethnically diverse groups and to examine whether experiences of the intervention and trial procedures differ across groups."*

16. • The manuscript would benefit from the inclusion of more recent qualitative research (2022–2025) focusing on patients' experiences of seeking care for self-harm, as well as factors influencing acceptability and engagement with services. While the current references provide a strong epidemiological foundation, they place less emphasis on patient perspectives, which are central to the stated aims of the study. Incorporating up-to-date qualitative and patient-centred studies would strengthen the contextualization of the findings and improve alignment with the study's objectives.

We have updated the manuscript to incorporate recent qualitative, patient-centred research on

experiences of seeking care for self-harm, strengthening the contextualisation of our findings and alignment with the study aims [Page 15].

"Recent qualitative research highlights that individuals who seek care following self-harm often emphasise the importance of feeling listened to, validated, and treated with compassion, with negative care experiences shaping future help seeking and engagement with services.33,34 Studies exploring brief psychological and crisis based interventions also suggest that perceived acceptability and engagement are closely linked to relational factors, such as trust, collaborative working, and continuity of care.35,36 These findings provide important context for interpreting participants' accounts in the present feasibility study and reinforce the centrality of patient experience in evaluating interventions for self-harm."

VERSION 2 - REVIEW

**Reviewer**	**1**
**Name**	**Furqan, Zainab**
**Affiliation**	**University of Toronto, Department of Psychiatry**
**Date**	**14-May-2026**
**COI**	

The authors have done a commendable job in addressing the feedback.

Most importantly, the methodology has been strengthened and clarified and appropriate caution in interpretation of findings has been recommended.

There are a few minor revisions and this reviewer would encourage authors to improve typos and readability. With these minor modifications, I believe the manuscript would be suitable for publication.

In the abstract, the authors write: "Participants were positive about the intervention"

I would recommend re-wording this to "participants generally expressed positive views about the intervention"

The last sentence is: "Interviews suggest that COPESS may be feasible and acceptable for delivery in primary care settings and warrant further evaluation in a full efficacy trial."

I would recommend re-wording to "Study findings suggest..."

VERSION 2 - AUTHOR RESPONSE

Reviewer: 1
Comments to the Author:

2. The authors have done a commendable job in addressing the feedback. Most importantly, the methodology has been strengthened and clarified and appropriate caution in interpretation of findings has been recommended. There are a few minor revisions, and this reviewer would encourage authors to improve typos and readability. With these minor modifications, I believe the manuscript would be suitable for publication.

> We thank the reviewer for their previous helpful suggestions and their positive comments on the revised manuscript. We are pleased that the methodological revisions and more cautious interpretation have addressed the main concerns raised in the previous review. We have carefully reviewed the manuscript and made further minor edits in line with reviewer suggestions to improve clarity and flow.

3. In the abstract, the authors write: "Participants were positive about the intervention". I would recommend re-wording this to "participants generally expressed positive views about the intervention"

> This has been amended as suggested [**Page 2**]

4. The last sentence is: "Interviews suggest that COPESS may be feasible and acceptable for delivery in primary care settings and warrant further evaluation in a full efficacy trial." I would recommend re-wording to "Study findings suggest..."

> This has been amended as suggested [**Page 18**]
